# Thymosin α1: a novel therapeutic option for patients with refractory chronic purulent rhinosinusitis

**DOI:** 10.1186/2045-7022-3-S2-P23

**Published:** 2013-07-16

**Authors:** Virgil Dalm, Rochus Neeleman, Harm de Wit, Hemmo Drexhage

**Affiliations:** 1 Erasmus MC, Immunology, Rotterdam, the Netherlands

## Background

Chronic purulent rhinosinusitis (CPR) is an inflammatory disorder of the nose and paranasal sinuses of unknown cause. Despite various available medical and surgical treatment options still 5 to 10% of patients remain refractory. Immune deficiency is one of the underlying risk factors for CPR and previous studies demonstrated defects in monocyte chemotaxis. Subsequent treatment with the thymic hormone preparation thymostimulin led to in vitro restoration of monocyte chemotaxis and significant clinical improvement in patients. However, thymostimulin became unavailable in recent years. In the present study we evaluated the effects of the thymic peptide thymosin α1 on monocytes from CPR patients as well as aberrant gene expression profiles in these monocytes in order to further elucidate the pathogenesis of CPR.

## Method

Monocytes were isolated from 16 patients with CPR and 13 healthy volunteers. Monocyte polarization was assessed using the Cianciolo and Snyderman monocyte polarization assay and the effects of thymosin α1 on monocyte polarization were evaluated. Furthermore, by Affymetrix whole-genome gene expression profiling and Q-PCR analysis we analyzed aberrant gene expression profiles in monocytes from CPR patients.

## Results

In 4 out of 16 CPR patients we found diminished monocyte polarization (45%, sd 8%) when compared to healthy volunteers (58%, sd 12,5%)(p=0.078). More interestingly, in vitro treatment with thymosin α1 significantly restored monocyte polarization in these patients (64% sd 10%,p=0.029). In the "poor polarizing" monocytes we found aberrant expression of genes involved in pathways of inflammation, chemotaxis and cell migration.

## Conclusion

Patients with CPR show diminished monocyte polarization in vitro that could be restored by thymosin α1. Moreover, these monocytes show an aberrant gene expression profile. We hypothesize that thymosin α1 may be a promising agent in treatment of refractory CPR patients. These effects may be mediated through interference with pathways involving the aberrantly expressed genes.

